# Pdr18 is involved in yeast response to acetic acid stress counteracting the decrease of plasma membrane ergosterol content and order

**DOI:** 10.1038/s41598-018-26128-7

**Published:** 2018-05-18

**Authors:** Cláudia P. Godinho, Catarina S. Prata, Sandra N. Pinto, Carlos Cardoso, Narcisa M. Bandarra, Fábio Fernandes, Isabel Sá-Correia

**Affiliations:** 1 IBB - Institute for Bioengineering and Biosciences, Department of Bioengineering, Instituto Superior Técnico, Universidade de Lisboa, Av. Rovisco Pais, 1049-001 Lisbon, Portugal; 2 Centro de Química-Física Molecular, Institute of Nanoscience and Nanotechnology, Instituto Superior Técnico, Universidade de Lisboa, Av. Rovisco Pais, 1049-001 Lisbon, Portugal; 3 DivAV, IPMA - Instituto Português do Mar e da Atmosfera, Rua Alfredo Magalhães Ramalho 6, 1495-006 Lisbon, Portugal

## Abstract

*Saccharomyces cerevisiae* has the ability to become less sensitive to a broad range of chemically and functionally unrelated cytotoxic compounds. Among multistress resistance mechanisms is the one mediated by plasma membrane efflux pump proteins belonging to the ABC superfamily, questionably proposed to enhance the kinetics of extrusion of all these compounds. This study provides new insights into the biological role and impact in yeast response to acetic acid stress of the multistress resistance determinant Pdr18 proposed to mediate ergosterol incorporation in plasma membrane. The described coordinated activation of the transcription of *PDR18* and of several ergosterol biosynthetic genes (*ERG2-4*, *ERG6*, *ERG24*) during the period of adaptation to acetic acid inhibited growth provides further support to the involvement of Pdr18 in yeast response to maintain plasma membrane ergosterol content in stressed cells. Pdr18 role in ergosterol homeostasis helps the cell to counteract acetic acid-induced decrease of plasma membrane lipid order, increase of the non-specific membrane permeability and decrease of transmembrane electrochemical potential. Collectively, our results support the notion that Pdr18-mediated multistress resistance is closely linked to the status of plasma membrane lipid environment related with ergosterol content and the associated plasma membrane properties.

## Introduction

**Figure 1:**
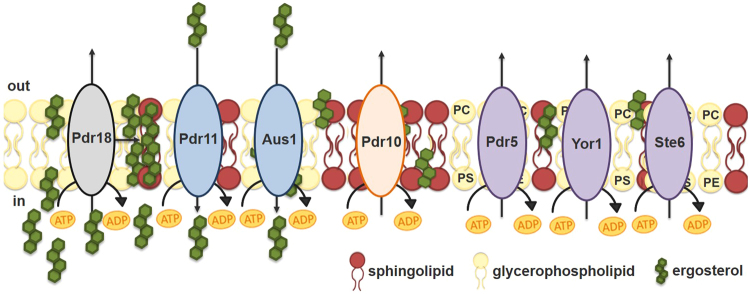
Plasma membrane ABC transporters of *S. cerevisiae* involved in lipid trafficking. Schematic representation of a set of ABC transporters that localize to the plasma membrane and are documented to play physiological roles in lipid trafficking and homeostasis in *S. cerevisiae*^7,8,16–21^. Pdr18, Pdr11, Aus1, Pdr10 and Pdr5 belong to the pleiotropic drug resistance (PDR) subfamily, Yor1 belongs to the multidrug resistance-associated protein (MRP) subfamily and Ste6 belongs to the multidrug resistance (MDR) subfamily of ABC transporters. The proposed biological functions are detailed in the Introduction section. Ergosterol transport, from its site of synthesis to the plasma membrane, is considered to occur by equilibration (t_1/2_ ~ 10–15 min) of endoplasmic reticulum and plasma membrane ergosterol pools via a bidirectional, nonvesicular process^66^. Abbreviations: phosphatidylcholine (PC), phosphatidylserine (PS), phosphatidylethanolamine (PE).

The acquisition of multidrug/multixenobiotic resistance (MDR/MXR) is widespread in nature and has clinical, agricultural and biotechnological implications. MDR/MXR transporters that are presumably able to catalyze the efflux of multiple cytotoxic compounds play a role in this phenomenon^1–5^. Two of the described MDR/MXR transporter families occur in all classes of organisms and belong to the ATP binding cassette (ABC) superfamily, which uses the hydrolysis of ATP to translocate a variety of solutes across biological membranes^2,3^, or to the Major Facilitator Superfamily (MFS) which energetically drive their transport utilizing the transmembrane electrochemical gradient^1,4^. Although those proteins have been traditionally considered drug exporters, their physiological function and the exact mechanism of their involvement in resistance to cytotoxic compounds are still unclear and it is puzzling that a wide range of structurally and functionally unrelated substrates may be exported by a specific transporter. Moreover, numerous ATP-binding cassette (ABC) transporters implicated in MDR/MXR are present in yeast genomes, strongly suggesting that they might have important physiological roles even in the absence of drugs/xenobiotics^3^. In fact, recent studies support the concept that at least some MDR/MXR transporters exert their effect as the result of a natural physiological role in the cell, rather than through the direct export of cytotoxic compounds^1,6–8^. In particular, the role of ABC-MDR/MXR transporters in yeast plasma membrane lipid homeostasis is gaining increasing attention^9^ and can also explain the MDR/MXR phenotype exhibited by cells expressing these transporters. Indeed, the maintenance of plasma membrane integrity is crucial in yeast cell tolerance to stress and lipids play a critical role in determining membrane physical properties and regulating the function of membrane associated proteins^10–15^. The *Saccharomyces cerevisiae* ABC transporters demonstrated in the scientific literature as involved in lipid trafficking and membrane lipid homeostasis are shown in Fig. 1. For example, Pdr11 and Aus1 are responsible for the uptake of exogenously supplied ergosterol in anaerobic conditions when ergosterol biosynthesis is impaired^16–18^. Also, the ABC efflux pumps Pdr5, Yor1 and Ste6 are implicated in the translocation of phospholipids between the two plasma membrane monolayers, thus contributing to plasma membrane asymmetry^19,20^. Pdr10 is associated with detergent-resistant domains in the plasma membrane and affects lipid distribution and maintenance of membrane micro-environment for the adequate functioning of membrane embedded proteins including the ABC-MDR transporter Pdr12^21^. Pdr16 and Pdr17 do not localize to the plasma membrane and their expression was proposed to regulate lipid metabolic pathways^22–24^ but the underlying mechanisms were not detailed.

The present work focuses on the yeast ABC transporter Pdr18, previously described as a MDR/MXR determinant and proposed to mediate ergosterol incorporation in yeast plasma membrane^7,8^, Pdr18 expression confers increased yeast resistance to the herbicides 2,4-dichlorophenoxyacetic acid (2,4-D), 2-methyl-4-chlorophenoxyaceticacid (MCPA), and barban, the agricultural fungicide mancozeb, the metal cations Zn^2+^, Mn^2+^, Cu^2+^ and Cd^2+ ^^7^, and to ethanol^8^. *PDR18* expression was found to contribute to reduce [^14^C]-2,4-D intracellular accumulation but its action may be indirect since 2,4-D induces the decrease of plasma membrane ergosterol content, this deleterious effect being much stronger in the *pdr18*Δ background^7^.

In this article, we describe Pdr18 role in yeast adaptation and tolerance to acetic acid stress. Acetic acid is widely used by the food industry as preservative of acidic food and beverages but there are spoilage yeasts that are able to cope with standard levels causing severe economic losses^25^. Acetic acid tolerance may however be a highly desirable trait in industrial yeasts, since this weak acid is an industrial fermentation byproduct, its accumulation in the medium contributing to reduced fermentation performance^26,27^. Moreover, acetic acid is present in lignocellulosic biomass hydrolysates for the production of bioethanol and other bio-based chemicals^28,29^. Therefore, the understanding of the mechanisms underlying the toxic effects and adaptation and tolerance to acetic acid in the biotechnologically-relevant yeast species *S. cerevisiae* is essential to guide more efficient strategies either for food spoilage control or for the development of more robust industrial strains^30–36^.

Yeast adaptation to weak acids is a complex multifactorial process considered to include mechanisms leading to the reduction of the intracellular concentration of the counter-ions through their increased efflux from the cell interior^31^ and restriction of the diffusional entry of the liposoluble acid form into the cell^31,36,37^. In particular, incorporation of a higher fraction of sphingolipids was found to improve membrane bilayer thickness and density, leading to the decrease of plasma membrane non-specific permeability in the highly acetic acid resistant species *Zygosaccharomyces bailii*^37^. Interestingly, the sphingolipid biosynthetic pathway was found to be up-regulated in response to acetic acid-induced stress^38^. Also, plasma membrane enrichment in saturated acyl chains arising from glycerophospholipids and complex sphingolipids was also found to occur in response to acetic acid stress and lead to reduced cell permeability and increased membrane order^39,40^. A chemogenomic study has identified in *S. cerevisiae* several acetic acid resistance determinant genes encoding ergosterol biosynthetic enzymes^30^ and the inclusion of a higher content of sterols in yeast plasma membrane demonstrated to counteract the deleterious effects of ethanol^41–45^ and heat shock^46,47^. The content of ergosterol is not only crucial for plasma membrane stability and adequate selective permeability barrier to avoid the passive diffusion of toxic compounds into the cell^48,49^, but is also related with the formation of lipid-raft domains that may modulate the activity of membrane-embedded pumps^50,51^.

The present study provides new insights into the biological role and impact of Pdr18 in yeast cell response under stress, with a focus on acetic acid stress. The observed coordinated activation of the transcription of *PDR18* and of several ergosterol biosynthetic genes during the period of adaptation to acetic acid inhibited growth provides further support to the involvement of Pdr18 in yeast response to maintain ergosterol content in plasma membrane of yeast stressed cells. The observed impact that such biological role has in counteracting acetic acid-induced decrease of plasma membrane lipid order, increase of the non-specific permeability and decrease of the transmembrane electrochemical potential establishes a link between this ABC transporter biological activity and its involvement in multistress resistance.

## Results

### *PDR18* gene expression is required for maximum tolerance to acetic acid

**Figure 2:**
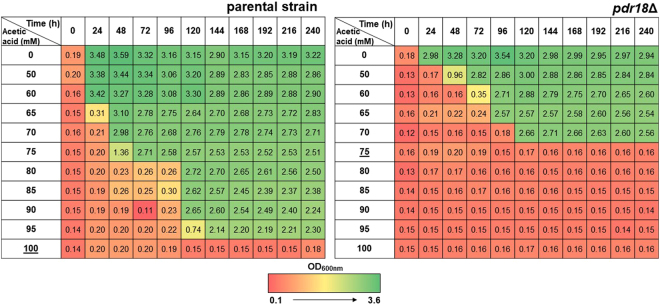
Inhibitory effect of acetic acid in the parental strain and *pdr18*Δ mutant growth. Growth was assessed based on culture OD_600nm_ during 240 hours of incubation in liquid MM4 supplemented with increasing concentrations of acetic acid (at pH 4.0). The minimum inhibitory concentration (MIC) found for the parental strain and *pdr18*Δ mutant (100 or 75 mM acetic acid, respectively) is underlined. Results are representative of three independent growth experiments.

**Figure 3:**
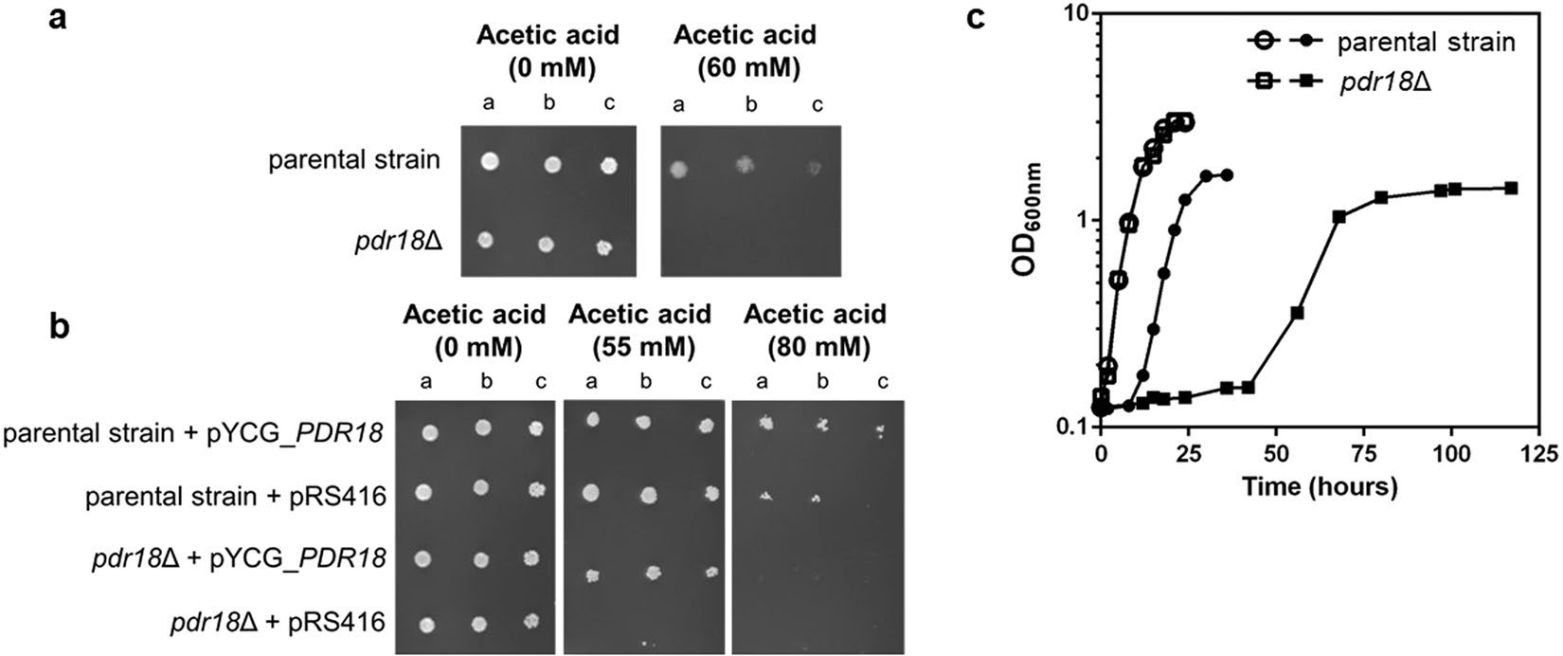
*PDR18* gene is a determinant of acetic acid tolerance in yeast. (**a**) Comparison of growth by spot assays of the parental strain and *pdr18*Δ mutant cell suspensions plated in solid MM4 media supplemented or not with acetic acid (at pH 4.5). (**b**) Complementation assays of growth in solid MM4-U media of the parental and *pdr18*Δ strains, harboring plasmid pRS416 (cloning vector) or the recombinant vector expressing *PDR18* from its natural promoter. In (**a**) and (**b**) the cell suspensions used to prepare the spots in lanes b) and c) were 1:5 and 1:25 serial dilutions, respectively, of the cell suspensions with an OD_600nm_ = 0.05 ± 0.005 in lane a) prepared from an exponentially growing culture of each strain. (**c**) Growth curves of the parental (○, ●) and *pdr18*Δ (□, ■) strains in MM4 liquid medium supplemented (●, ■) or not (○, □) with 60 mM acetic acid at pH 4.0 based on culture OD_600nm_. Results of all panels of the figure are representative of at least three independent growth experiments.

The expression of the ATP-binding cassette plasma membrane transporter encoding gene *PDR18* was found to be required to partially overcome the effects of acetic acid induced stress in yeast growth (Figs 2 and 3). Susceptibility towards this weak acid was assessed based on yeast growth in liquid medium by comparing the growth of a parental strain *Saccharomyces cerevisiae* BY4741 and the derived deletion mutant *pdr18*Δ exposed to a wide range of acetic acid concentrations and determining the minimum inhibitory concentration (MIC) (Fig. 2). While the parental strain was able to grow in the presence of concentrations equal or below 95 mM, but not in the presence of 100 mM acetic acid (the calculated MIC for this strain), the MIC for the deletion mutant *pdr18*Δ was 75 mM (Fig. 2). Moreover, the final biomass attained after yeast cultivation under acetic acid stress showed a dose-dependent decrease and was more evident for the *pdr18*Δ mutant (Fig. 2).

Spot assays in agar plates of MM4 medium supplemented with 60 mM of acetic acid (at pH 4.5) confirmed the role of Pdr18 as an acetic acid resistance determinant (Fig. 3a). In fact, the susceptibility phenotype of the *pdr18*Δ mutant under acetic acid was rescued by the insertion of a centromeric plasmid expressing the *PDR18* gene under the control of its natural promoter. Moreover, the expression of Pdr18 from the recombinant plasmid in the parental strain improved its resistance to acetic acid (Fig. 3b).

Most of the experiments described in this work were performed using the standardized concentration of acetic acid (60 mM, at pH 4.0). Under these conditions, growth curve of the parental strain in acetic acid supplemented MM4 medium exhibit a lag-phase of approximately 10 hours that was not detectable in the absence of acetic acid stress, while the duration of the acetic acid-induced lag-phase was extended to approximately 40 hours for the *pdr18*Δ mutant (Fig. 3c; Supplementary Fig. S1). Also, the maximum specific growth rates and the final biomass concentrations attained were more drastically reduced on the mutant compared to the parental strain under acetic acid stress (Fig. 3c).

### *PDR18* and *ERG*-genes transcription is up-regulated in response to acetic acid stress contributing to the maintenance of plasma membrane ergosterol content

**Figure 4:**
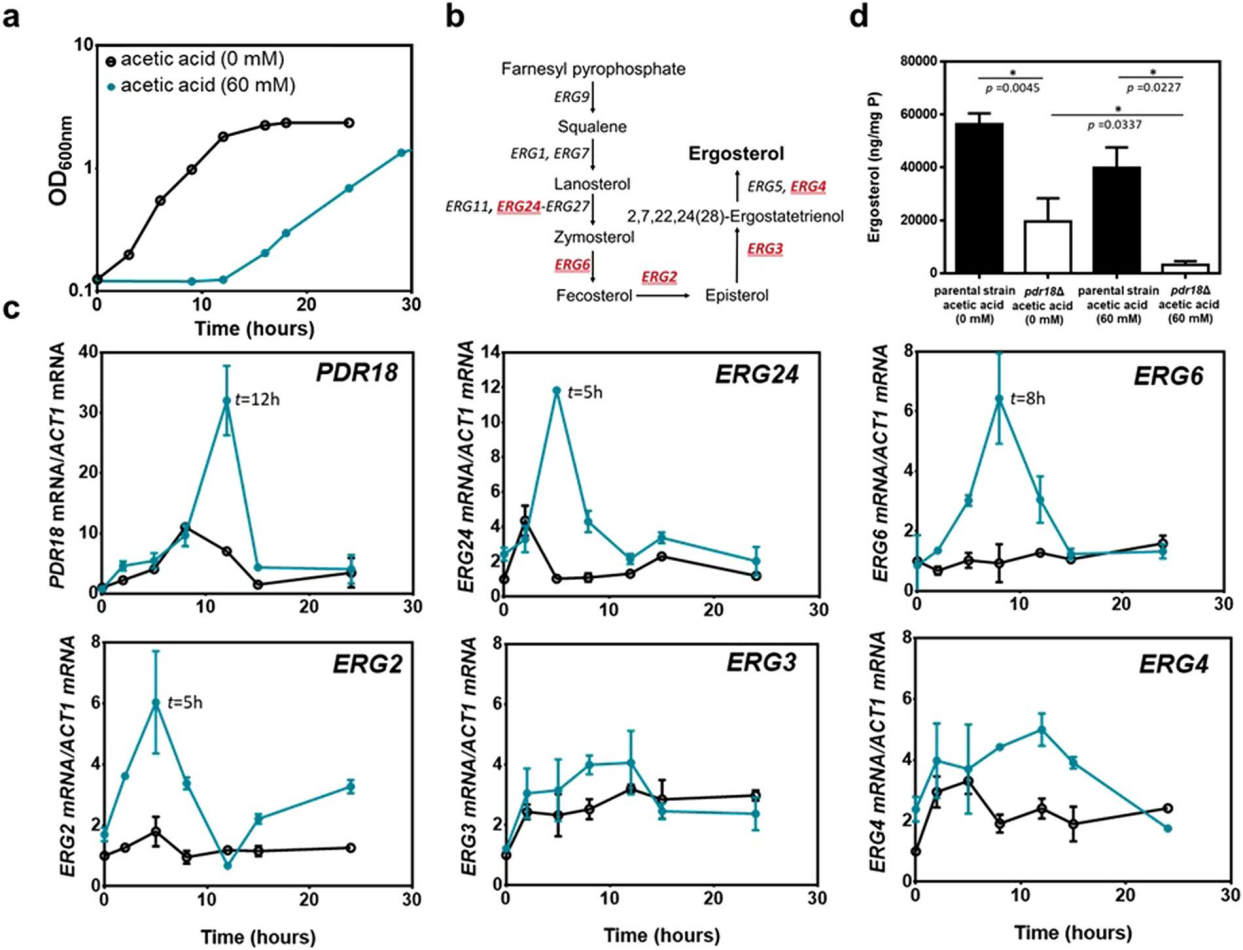
Levels of mRNA from *PDR18* and from several ergosterol biosynthetic pathway genes during cultivation in absence or presence of acetic acid and comparison of ergosterol levels. (**a**) Growth curve of *S. cerevisiae* BY4741 in absence (○) or presence () of 60 mM acetic acid (at pH 4.0) based on culture OD_600nm_. (**b**) Ergosterol biosynthetic pathway [adapted from KEGG (http://www.genome.jp/kegg/pathway.html)]; the *ERG*-genes under study are highlighted in red; (**c**) mRNA levels from the indicated genes by qRT-PCR, using *ACT1* as the reference gene. (**d**) Ergosterol content in cell membranes from exponentially-growing cells of the parental and *pdr18*Δ strains in the absence or presence of 60 mM acetic acid (60 mM). Error bars represent standard deviation resultant from at least two biological replicates with three technical replicates each.

Levels of mRNA from *PDR18* and selected *ERG*-genes in *S. cerevisiae* BY4741 cells were compared during cultivation of cells not previously adapted to acetic acid in MM4 or MM4 supplemented with 60 mM acetic acid at several time-points (Fig. 4). Results show that *PDR18* transcription is activated in response to acetic acid stress. Maximum activation levels occur when the cell population resumes growth after an extended period of adaptation to growth under acetic acid stress and these levels were 3-fold higher than in unstressed cells. However, during exponential growth in the presence of acetic acid, mRNA levels from *PDR18* decreased to values close to those in unstressed cells. In the absence of stress, the transcription profile of *PDR18* is also dependent on the growth phase, mRNA levels peaking during exponential growth (Fig. 4).

Since several enzymes catalyzing post-squalene steps of the ergosterol biosynthetic pathway have been related with acetic acid stress response^30,52^, the transcription profiles of *ERG2-4*, *ERG6*, and *ERG24* genes were obtained. These genes are known determinants of resistance to acetic acid/weak acid stress in yeast^30,31,53^. The transcription levels from those *ERG* genes during acetic acid stressed growth show two profiles: *ERG24*, *ERG6* and *ERG2* have a peak of activation (6–12 fold) after 5–8 hours, by the mid- to end of the phase of latency, while for *ERG3* and *ERG4* genes the activation levels are lower (2–5-fold) and were maintained throughout cultivation (Fig. 4). Ergosterol quantification confirms its lower content in membranes of exponentially-growing unstressed cells of the deletion mutant *pdr18*Δ compared to the parental strain. The ergosterol content in *pdr18*Δ cells exponentially-growing under acetic acid stress was found to be dramatically reduced (approximately 10-fold), compared to parental strain levels cells growing in the same conditions (Fig. 4). These results confirm the need of Pdr18 expression to maintain ergosterol content in plasma membrane especially under acetic acid stress. Collectively, these results are consistent with the biological function attributed to Pdr18 in ergosterol homeostasis and indicate that under acetic acid stress *PDR18* gene expression regulation is part of the yeast response to acetic acid stress together with the activation of ergosterol biosynthesis.

### *PDR18* expression leads to a more ordered plasma membrane especially under acetic acid stress

As ergosterol content is an important factor affecting yeast plasma membrane order^42,54,55^, the next step was to assess plasma membrane order in the parental and *pdr18*Δ cell strains grown either unstressed or in the presence of acetic acid stress to further understand the impact that *PDR18* expression has at the level of plasma membrane properties.

Laurdan is a membrane dye whose fluorescence emission spectrum depends on lipid packing. For more ordered membranes, the emission spectrum of Laurdan is shifted to lower wavelengths relative to the spectrum of the same probe in disordered membranes. This spectral shift can be quantified through the calculation of a generalized polarization (GP) factor from the fluorescence intensity of two spectral channels of Laurdan emission spectra^56^. The GP value of Laurdan is a convenient and quantitative parameter to evaluate membrane packing, with higher values reflecting higher ordering.

**Figure 5:**
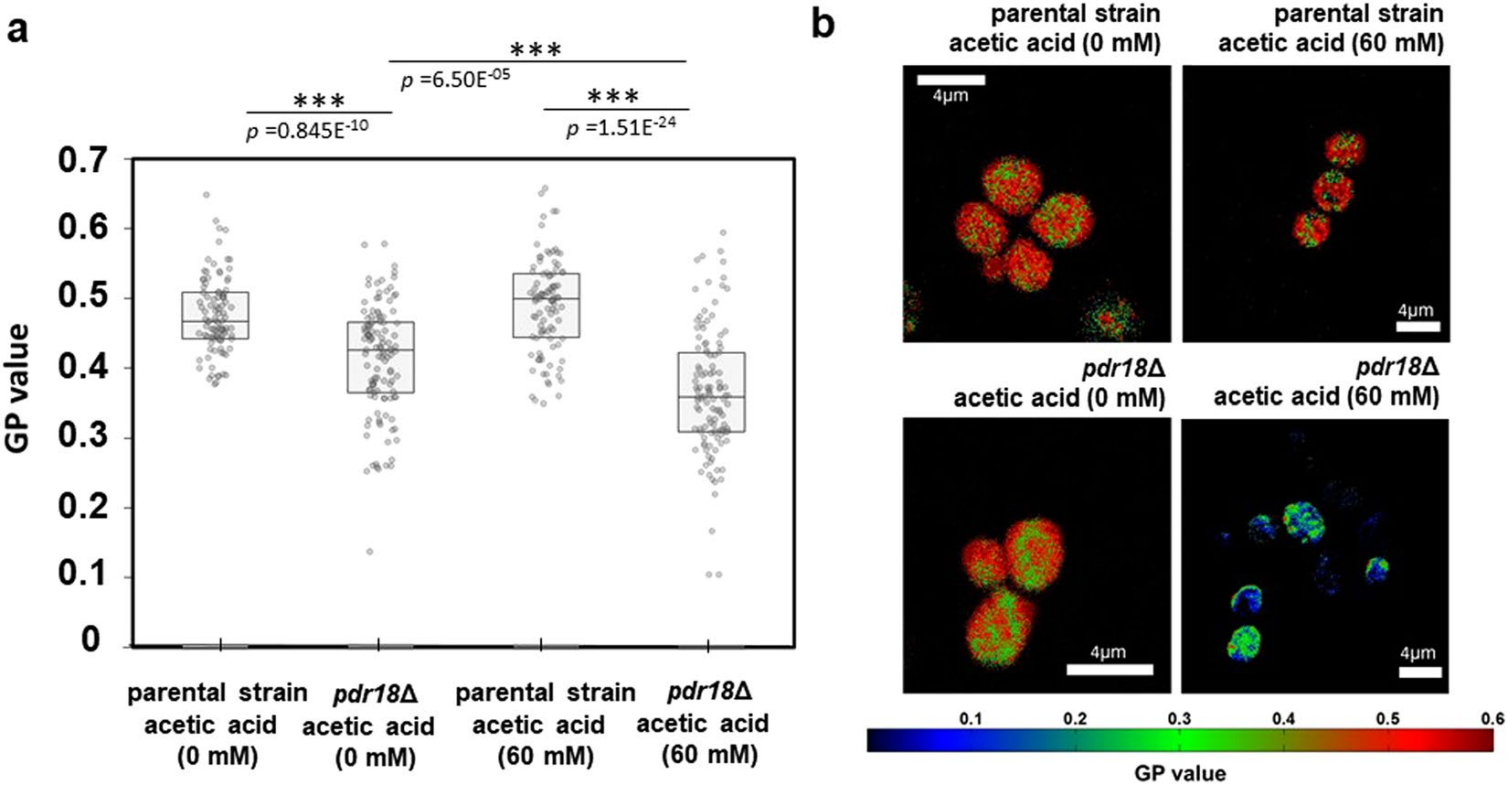
Plasma membrane order of the parental and *pdr18*Δ strains growing in absence or presence of acetic acid, as reported by Laurdan Generalized Polarization (GP) values. (**a**) GP values from individual exponentially-growing cells of the parental and *pdr18*Δ strains cultivated either in the presence or absence of 60 mM acetic acid at pH 4.0. The boxplots represent the first and third quartiles and median for each cell population; (**b**) Examples of Laurdan Generalized Polarization images for the parental strain and *pdr18*Δ cells grown in absence or presence of acetic acid stress. Results arise from the analysis of at least 100 cells obtained from three independent assays. Asterisks represent statistical significant differences, based on t-student tests.

The distribution of Laurdan Generalized Polarization (GP) values for each individual cell is shown in Fig. 5a and illustrative examples of GP images are shown in Fig. 5b. The parental strain cell population harvested in the exponential phase of growth show a significantly higher plasma membrane order than the population of cells lacking *PDR18* gene, when both strains were grown either in the absence (*p* = 0.845E^−10^) or in presence (*p* = 1.51E^−24^) of 60 mM acetic acid at pH 4.0 (Fig. 5a). Parental cells harvested in the exponential phase of growth without acetic acid or adapted to that concentration of acetic acid show no significant difference in plasma membrane order, as assessed by the GP mean value (Fig. 5a). However, Laurdan GP values, reflecting plasma membrane order, of *pdr18*Δ cells decreased by 12% when cultivated in the presence of acetic acid (Fig. 5a). Also noteworthy is that Laurdan GP values for the *pdr18*Δ cell population exhibit an interquartile range (Q3-Q1) 53% larger than the parental strain population, both grown in the absence of acetic acid, which indicates a higher cell heterogeneity for plasma membrane order within the *pdr18*Δ cell population (Fig. 5a). Adapted cells exponentially-growing in the presence of acetic acid that lack the *PDR18* gene, exhibit a lower plasma membrane order (25% lower GP mean value) and a more heterogeneous profile for this trait (25% larger interquartile range), compared with the stressed parental strain population (Fig. 5a).

### Acetic acid stress induces an increase in plasma membrane permeability when yeast cells lack *PDR18*

**Figure 6:**
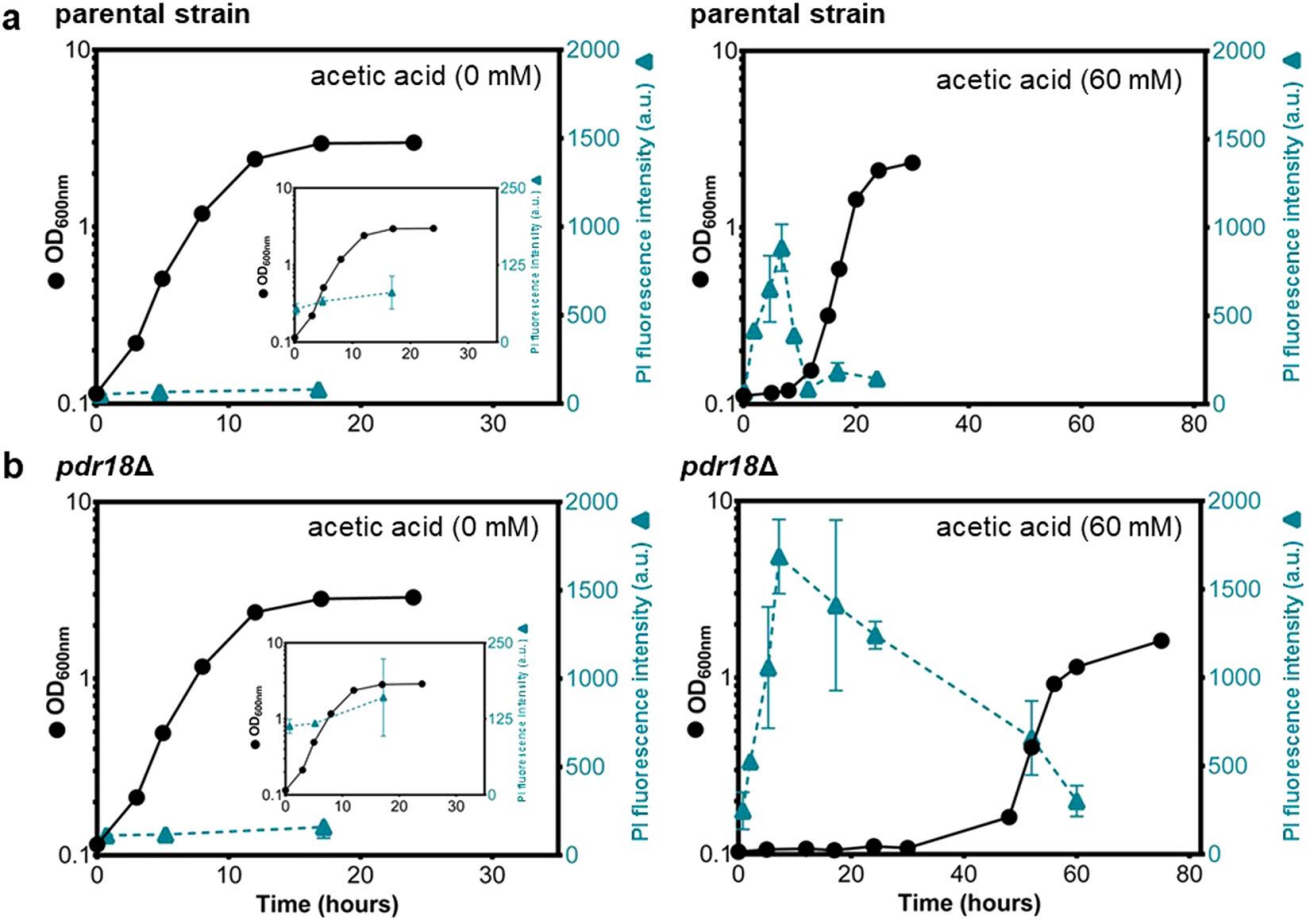
Plasma membrane permeability of the parental strain and *pdr18*Δ cells during the growth curve in the presence or absence of acetic acid. Comparison of plasma membrane permeability (; arbitrary units) during cultivation of the parental (**a**) or *pdr18*Δ (**a**) strains in the presence or absence of 60 mM acetic acid at pH 4.0. The estimation of plasma membrane permeability is based on the fluorescence intensity values exhibited by yeast cells upon passive accumulation of propidium iodide. Graphs with a more appropriate fluorescence intensity scale are shown to allow comparison between (**a**) and (**b**) in the absence of acetic acid. Error bars represent standard deviation (n = 3).

**Figure 7:**
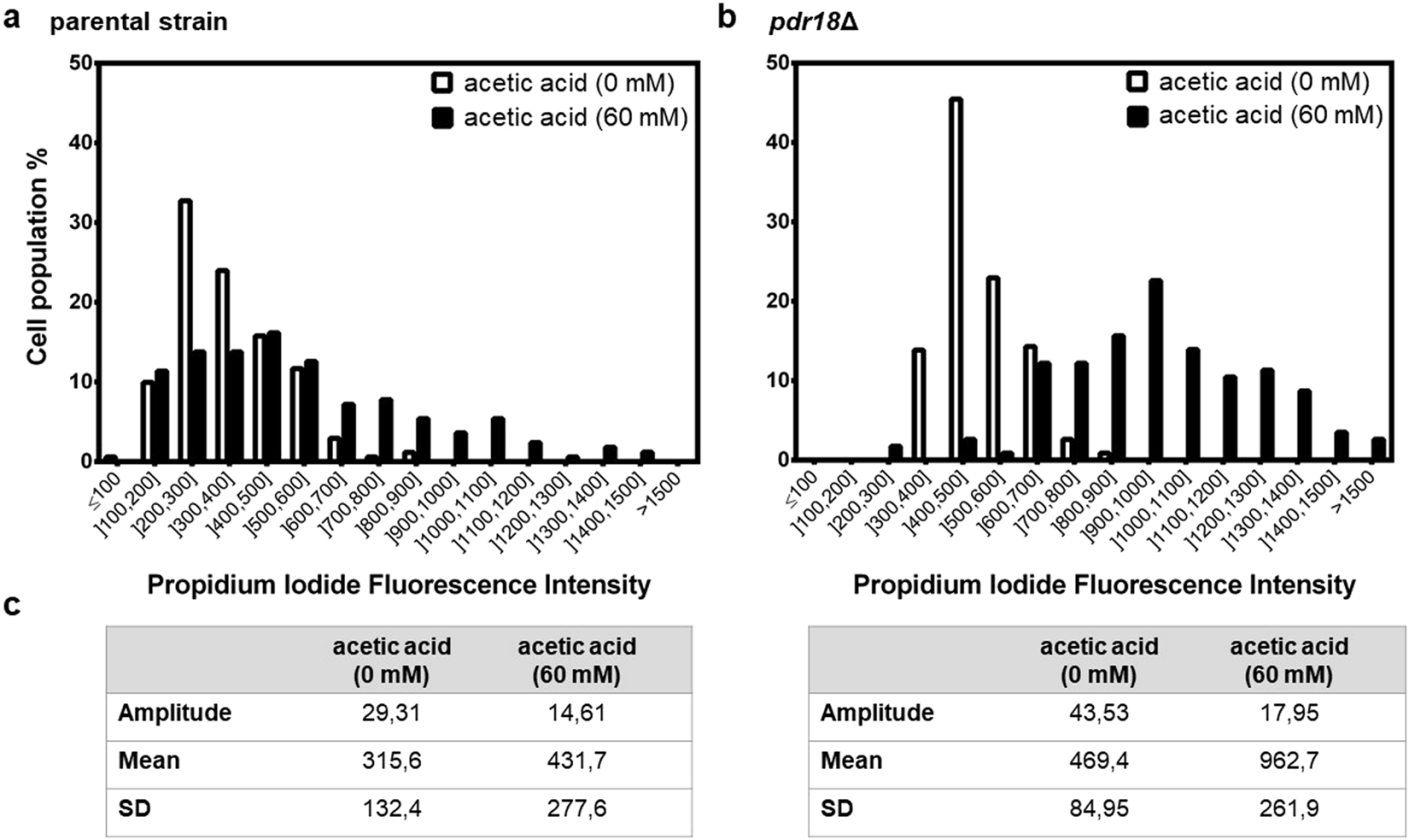
Cell-by-cell propidium iodide fluorescence in exponentially-growing cell populations of parental or *pdr18*Δ strain in the presence or absence of acetic acid. Distribution of propidium iodide fluorescence intensity classes of membrane permeability of the parental strain (**a**) and *pdr18*Δ (**b**) cell population harvested in the exponential phase in absence (□) or presence (■) of 60 mM acetic acid; (**c**) Statistical parameters of a Gaussian curve fit to the respective data shown in (**a**) and (**b**).

The less ordered plasma membrane lipid environment of the *pdr18*Δ deletion mutant assessed before was found to have a considerable impact on the physiological function of yeast plasma membrane as a selective barrier in the presence of acetic acid, with plasma membrane permeability being inferred based on propidium iodide (PI) passive uptake (Figs 6 and 7). PI is a cell-impermeant dye with a high affinity for nucleic acids. The dye only penetrates cells with compromised plasma membranes; the subsequent binding to intracellular nucleic acids increases PI fluorescence up to 30-fold^57^. Therefore, the PI fluorescence intensity parameter can be correlated with the proportion of permeabilized cells.

Even in the absence of any stress, cells deleted for *PDR18* are more permeable than cells of the parental strain harvested during the growth curve (25% higher permeability; Fig. 6). Acetic acid supplementation of the growth medium (60 mM at pH 4.0) leads to an increased plasma membrane permeability in parental strain cells during the growth curve, PI uptake values peaking at 7 hours (10-fold the levels in absence of acetic acid) by the time when cell population resumes growth after the adaptation phase (Fig. 6a). Remarkably, during exponential growth under acetic acid stress, yeast cell permeability is reduced, and cells harvested at mid-exponential phase exhibit a permeability value similar (only 1.3-fold higher) to unstressed cells (Fig. 6a). In *pdr18*Δ mutant cells, plasma membrane permeability peaks during acetic-acid induced lag-phase, reaching values above those registered for the parental strain and much higher (15-fold) than those registered for unstressed cells (Fig. 6b). Even during exponential growth adapted to acetic acid, the permeability of cells deleted for *PDR18* gene is above the values for unstressed cells (4-fold higher) and this cell population was unable to recover from acetic acid-induced permeabilization during lag-phase to levels similar to unstressed cells (Fig. 6b).

Analysis of fluorescence levels of individual cells allow the comparison of exponentially-growing cell populations fluorescence distributions (Fig. 7a,b). The distributions fit to a gaussian curve (Fig. 7c) and the acetic acid-adapted parental cell population exhibits a higher average PI fluorescence intensity and more heterogeneous cell-to-cell permeability levels [higher standard deviation (SD) and lower amplitude] than the unstressed cell population (Fig. 7). Deletion of *PDR18* gene leads to a fluorescence distribution in the cell population with a higher average value of plasma membrane permeability even for cells cultivated in the absence of acetic acid. Such heterogeneity increases under acetic acid stress (Fig. 7).

### *PDR18* gene expression is essential to maintain plasma membrane potential in absence or presence of acetic acid stress

**Figure 8:**
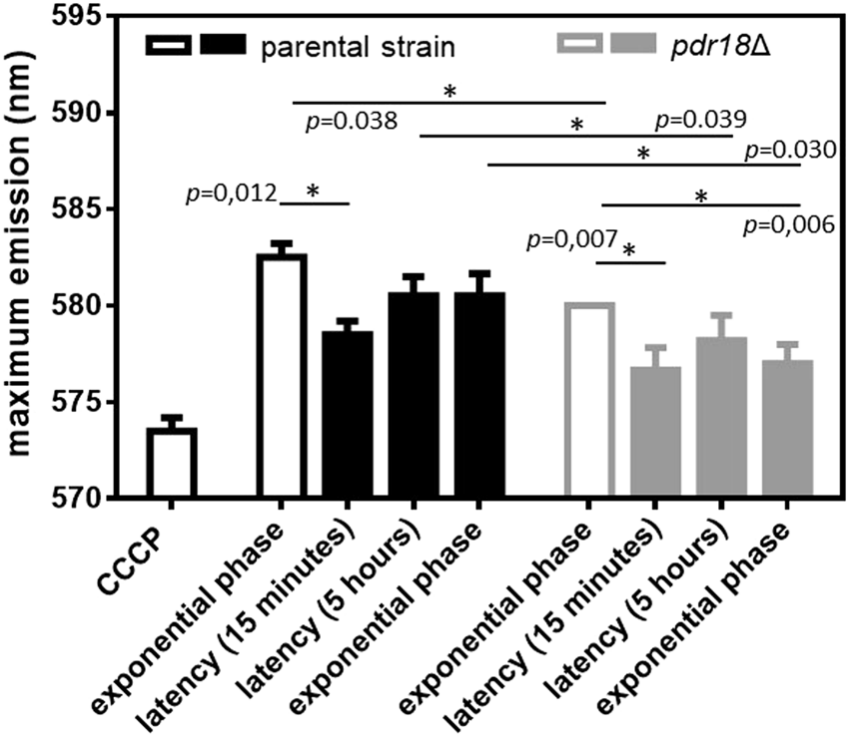
Plasma membrane electrochemical potential of the parental and *pdr18*Δ strains during cultivation in presence or absence of acetic acid. Comparison of plasma membrane electrochemical potential based on the maximum emission wavelength of the fluorescent probe DiSC_3_(3) in citrate-phosphate buffer at pH 6.0. Cells of parental (□, ■) and *pdr18*Δ (, ) strains were harvested at the exponential phase of growth in the absence of acetic acid (□, ) and at the indicated timepoints of the corresponding growth curves in the presence of 60 mM acetic acid (■, ). Error bars represent standard deviation resultant from at least two biological replicates with at least three technical replicates each. Asterisks represent statistical significance in t-student test. CCCP: Carbonyl cyanide *p*-chlorophenylhydrazone.

The effect of *PDR18* expression in plasma membrane electrochemical potential when yeast cells were cultivated in presence or absence of acetic acid was also estimated based on the position of the emission maximum at equilibrium of the diS-C_3_(3), a dye sensitive to membrane potential. Since this diS-C_3_(3) assay was used to compare two isogenic strains (parental cells and cells deleted for PDR18), the activity of other MDR-pumps is considered identical and the differences in λ_max_^eq^ believed to reflect differences in membrane potential^58^. Results indicate that even in the absence of stress, parental strain cells exhibit a significantly higher (*p* = 0.038) plasma membrane potential than *pdr18*Δ cells (Fig. 8), as previously observed using other methods^7^. Acetic acid induced a short-term decrease of plasma membrane potential for both the parental strain and *pdr18*Δ cells after 15 minutes of cultivation in the presence of acetic acid (Fig. 8). However, after five hours of cultivation in the presence of acetic acid, parental strain cells recovered to values that were not significantly different from unstressed cells, and these levels were maintained during exponential growth of adapted cells (Fig. 8). For *pdr18*Δ acetic acid-adapted cells such recovery was not observed (Fig. 8). The incubation of exponentially-growing parental strain cells for 15 minutes with carbonyl cyanide *p*-chlorophenylhydrazone (CCCP), used in this assay as a positive control, confirmed the disruption of membrane potential under these conditions (Fig. 8).

## Discussion

The yeast *Saccharomyces cerevisiae* has evolved numerous and diverse complex mechanisms that allow cells to grow and thrive in stressful environments. Among them are those related with the remodeling of lipid and protein composition of plasma membrane, an active interface between the cell interior and its environment that shields yeast cells against a potentially harmful environment^11,59^. The composition of phospholipids, sphingolipids, and sterols in the plasma membrane play a critical role in determining its physical properties, such as permeability and fluidity, and has a strong influence on the activity of the proteins associated to or embedded in the lipid bilayer^50,60–62^. In this work we demonstrate the importance of the plasma membrane ABC transporter Pdr18 as a determinant of resistance to acetic acid. Pdr18 was previously involved in resistance to multiple stresses presumably by increasing plasma membrane ergosterol content^7,8^. Ergosterol, the major sterol present in fungal plasma membrane, modulates its thickness, fluidity and permeability and regulates the activity of membrane associated transporters^44,45,47–49,54,63^. In the present work, *PDR18* expression was found to be essential to maintain maximum ergosterol content in yeast plasma membrane and to counteract acetic acid-induced decrease of ergosterol content and plasma membrane order and its non-specific permeability and electrochemical potential levels. This role for Pdr18 in the maintenance of adequate plasma membrane physical properties under acetic acid stress is essential for adequate physiological function of this important cell membrane as a selective barrier thus allowing the efficient import of nutrients and excretion of toxic metabolites, such as the counter-anion acetate, as well as the restriction of the diffusional entry of acetic acid.

The transcriptional activation under acetic acid stress of a number of genes of the ergosterol biosynthetic pathway has been revealed by transcriptomic analysis^64^. The *ERG* genes found to be activated under acetic acid stress are also required for tolerance to this weak acid^30,31,64^, reinforcing the importance of the ergosterol pathway in yeast adaptation and tolerance to acetic acid. The interconnection between the ergosterol pathway and the function of the multistress resistance determinant Pdr18 under acetic acid stress is also suggested by the data obtained in this study, for the transcription analysis of *PDR18* and several genes of the ergosterol biosynthetic pathway. Yeast growth under acetic acid stress was found to involve the activation of all these genes with a peak of gene transcripts during advanced acetic acid-induced lag phase although the time-course profiles do not coincide. Remarkably, the peak of the mRNA level from *PDR18* was observed later than the peak registered for the first genes of the pathway tested (*ERG24*, *ERG6* and *ERG2*), consistent with the proposed role for Pdr18 at the end of the ergosterol transport process of the newly synthesized sterols from the endoplasmic reticulum (ER) to the plasma membrane. Moreover, the activation level of the late genes of the ergosterol pathway tested (*ERG3* and *ERG4*) was more moderate and the activation profile less dependent of the growth phase. It would be important to know the sensors that perceive the fluctuations in the membrane environment of acetic acid stressed cells and transduce the signals to *PDR18* gene resulting into the compensatory changes in the ergosterol profile and how these processes are controlled.

Yeast cells synthesize ergosterol in the membrane of the endoplasmic reticulum via a cascade of coupled enzymatic reactions^65^. Ergosterol has then to be transported from the site of its synthesis to the plasma membrane and although this transport is known to be fast, the mechanism by which newly synthesized sterols are transported from the ER to the plasma membrane is not fully understood^66,67^. An essential role for vesicular transport pathways in transporting ergosterol, from its site of synthesis to the plasma membrane has been ruled out and ergosterol transport was proposed to occur by equilibration (*t*_1/2_ ∼ 10–15 min) of endoplasmic reticulum and plasma membrane ergosterol pools via a bidirectional, nonvesicular process^66^. To reconcile an equilibration process with the high ergosterol concentration present in plasma membrane, and based on the observation that a large fraction of ergosterol is found in the plasma membrane condensed with sphingolipids in membrane rafts that coexist with free sterol, Baumann *et al*.^66^ hypothesized that the concentration of free sterol is similar in the plasma membrane and endoplasmic reticulum and that only free (non-raft) sterol molecules have access to a nonvesicular transport pathway that connects the two organelles. Based on the results of the present study, we propose the involvement of the plasma membrane associated ABC transporter Pdr18 in the active ergosterol transport process at the plasma membrane level, allowing the high physiological ergosterol concentration present in this membrane.

Like other organisms, the yeast *S. cerevisiae* has the ability to acquire resistance to multiple drugs (MDR) or to multiple xenobiotic compounds (MXR), *i.e*. to become less sensitive to a broad range of chemically and functionally unrelated cytotoxic compounds^32,33,68–70^. The acquisition of resistance to multiple stressing agents is due to a number of mechanisms, among them the mechanism mediated by efflux pump proteins localized at the plasma membrane and belonging to either the ABC (ATP-binding cassette) superfamily or the MFS (major facilitator superfamily) and proposed to enhance the kinetics of extrusion of these compounds^1,2,4,71^. The presence of a large number of ABC and MFS transporters homologous to MDR/MXR characterized transporters in the genomes of yeasts and other organism strongly suggests that they may play important physiological roles even in the absence of the cytotoxic compounds. Accumulating evidence has shown that ABC proteins perform endogenous activities extending beyond their accepted role as drug exporters, among them the transport of lipids^9,61,72,73^ as schematized in Fig. 1. Although *PDR18* has a paralogous gene in *S. cerevisiae*, *SNQ2*^74^, no demonstration of the eventual involvement of Snq2 in lipid transport and homeostasis was so far reported. However, the affinity of Snq2 transporter for sterols was hypothesized due to its role in alleviating estradiol toxicity in *S. cerevisiae*^75^. Moreover, we were not able to identify any phenotype of resistance to acetic acid associated to *SNQ2* expression. The coordinate control of plasma membrane ergosterol composition by Pdr18 and the so far well accepted role of Pleiotropic drug resistance (PDR) transporters in drug/xenobiotic efflux activity (in this particular case, the putative role of Pdr18 in the active acetate efflux) is a possible model. However, based on the data gathered in this study, the involvement of Pdr18 in multistress resistance may merely result from its physiological role in ergosterol transport and proper distribution within plasma membrane proved to have a significant impact in plasma membrane properties, especially relevant under acetic acid stress. Apart from serving as substrates of certain ABC transporters, lipid molecules are also crucial molecular determinants that affect the trafficking and functioning of certain ABC transporter proteins^18,76–78^. Therefore, the role of Pdr18 in ergosterol homeostasis at the plasma membrane level is likely to affect the proper functioning of other efflux pumps of the ABC superfamily or the MFS, already proposed to be required for acetate efflux^31^, or the essential plasma membrane H^+^-ATPase activity^50,79^. Collectively, results support the notion that multistress resistance mediated by Pdr18 is closely linked to the status of plasma membrane lipid environment, specifically related with ergosterol content and the associated plasma membrane properties.

## Methods

### Strains, plasmids and growth conditions

*Saccharomyces cerevisiae* parental strain BY4741 (*MATa*, *his3Δ1*, *leu2Δ0*, *met15Δ0*, *ura3Δ0*) and the derived deletion mutant *pdr18*Δ were obtained from the EUROSCARF collection (http://web.uni-frankfurt.de/fb15/mikro/euroscarf), as well as plasmid pYCG_*PDR18*, expressing the *PDR18* gene from its natural promoter, and the corresponding cloning vector, pRS416, used for phenotypic complementation assays.

Yeast cells were cultivated at 30 °C with orbital agitation (250 rpm) in liquid minimal growth medium supplemented with the amino acids and the nucleotide to support growth of the auxotrophic strains (MM4). MM4 contained 1.7 g/L yeast nitrogen base without amino acids and ammonium sulphate (Difco, Michigan, USA), 20 g/L glucose (Merck, Darmstadt, Germany), 2.65 g/L (NH_4_)_2_SO_4_ (Panreac AppliChem, Connecticut, USA), 20 mg/L L-methionine, 20 mg/L L-histidine (both from Merck, Darmstadt, Germany), 60 mg/L L-leucine and 20 mg/L L-uracil (both from Sigma, Missouri, USA), adjusted to pH 4.0 with HCl. Solid medium was prepared by the addition of 20 g/L agar (Iberagar, Barreiro, Portugal) and the pH was set to 4.5 with HCl. Cells harboring the cloning vector pRS416 or derived plasmids were grown in the same medium lacking uracil supplementation (MM4-U medium) to maintain selective pressure.

### Susceptibility assays

Growth curves were obtained by inoculating a mid-exponential cell suspension in 100 mL flasks containing 50 mL MM4 pH 4.0 supplemented or not with acetic acid pH 4.0 (set with NaOH) at the desired concentration. The starting OD_600nm_ of the cultivation media was standardized at 0.1 ± 0.05. Growth with orbital agitation was followed at 30 °C by measuring OD_600nm_.

Yeast cell suspensions used for the spot assays were prepared from a mid-exponential cell culture grown in MM4 medium, diluted to an OD_600nm_ of 0.05 (lane a in the figures), followed by two serial dilutions of 1:5 each (lanes b and c). These cell suspensions were plated as 4 µL spots onto the surface of MM4 pH 4.5 solid medium, supplemented or not with acetic acid. Complementation tests were perfomed with yeast cells harboring either the empty vector pRS416 or the recombinant plasmid pYCG_*PDR18*. For the complementation tests, spot assays were prepared as described before and cell suspensions were spotted onto MM4-U agar plates that were incubated at 30 °C for 72 hours.

### Time-course transcription analysis of *PDR18* and *ERG*-genes

For gene transcription assays, BY4741 cells were harvested at adequate time points from liquid medium cultivations performed in the presence and absence of 60 mM acetic acid, as described above. Total RNA was extracted by the hot phenol method^80^. The real-time Reverse Transcription–PCR protocol used followed the manufacturer’s instructions (Applied Biosystems, California, USA) and the primers used for the amplification of each target cDNA were designed using the Primer Express software (Applied Biosystems, California, USA) (Supplementary Table S1). The RT–PCR reaction was carried out using a thermal cycler block (7500 Real-Time PCR System; Applied Biosystems, California, USA). The *ACT1* mRNA level was used as the internal control. The relative value obtained for each target gene at the initial time point (15 minutes of incubation following inoculation) under unstressed conditions was set as 1 and the remaining values are relative to that value.

### Ergosterol quantification in yeast cell membranes

For quantification of ergosterol in yeast cell membranes, the parental and *pdr18*Δ strains were cultivated in MM4 either in the presence or absence of acetic acid stress (60 mM at pH 4.0) as described above. Exponentially-growing cells were harvested by centrifugation (5000* g*, 5 minutes, 4 °C), resuspended in homogenization buffer containing 50 mM Tris/HCl (pH 7.5), 2.5 mM EDTA and a protease inhibitor cocktail (1 mM PMSF and 1 μg/mL each of leupeptin, pepstatin A and aprotinin). Cells were broken by vortex-mixing with glass beads (Glaperlon 0.40–0.60 mm). The cell membranes were recovered by centrifugation (1000 *g*, 10 minutes, 4 °C) to remove unbroken cells and finally the cell membranes were pelleted by ultracentifugation at 25 000 rev./min for 1 hour (rotor type SW41Ti, Beckman Coulter). The pelleted cell membranes were resuspended in a buffer containing 20 mM Tris/HCl (pH 7.5), 150 mM NaCl, 20% glycerol and protease inhibitors at the concentrations mentioned above, stored frozen and freeze-dried until sterol extraction. Sterol extraction involved addition of 250 μL 5-α-cholestane (internal standard, Sigma) in dichloromethane and a lipid extraction using 600 μL methanol (Merck) and 300 μL dichloromethane (Merck). Samples were homogenized for 60 seconds at 2–4 °C in a homogenizer (polytron 10–35 GT, Kinematica) at 10,000 rpm. Afterwards, 300 μL ultra-pure water and 300 μL dichloromethane were added. Samples were centrifuged (3000 *g*, 10 minutes, 4 °C). The lower phase was filtered through an anhydrous sodium sulfate (Merck) layer and solvent evaporated under N_2_ at 20 °C. Lipids were saponified with 500 μL of 2.5 N KOH (Merck) at 80 °C for 1 hour. Saponified lipids were extracted with 1 ml of saturated NaCl (Merck) and 5 ml hexane (Merck). Organic phase was evaporated under N_2_ at 20 °C. Derivatization was done with 100 μL N,O-bis(trimethylsilyl)trifluoroacetamide with trimethylchlorosilane (Sigma) at 80 °C for 12 hours. Derivatized sterols were analyzed using GC-MS (Scion 456 GC-SQ, Bruker) with a DB5 column (30 m × 0.2 mm, 0.20 μm). The carrier gas was helium (flow rate of 1 mL/min). Oven temperature was held at 120 °C for 1 min, increased to 300 °C at a rate of 2 °C/min, and held at 300 °C for 20 min. Injection and detector temperatures were 300 °C. Peak identification was based on retention time and comparison with external standards in the spectral database. Quantification was achieved through the internal standard peak area. Sterol concentration was normalized taking into account phosphate content in the freeze-dried samples.

### Assessement of membrane lipid order by Laurdan fluorescence

Membrane lipid order was estimated in exponentially-growing cells of parental and *pdr18*Δ strains either in the absence or presence of acetic acid, by the normalized ratio of Laurdan fluorescence emission measured in two channels. Growth curves were performed as described above and cells at mid-exponential phase (OD_600nm_ = 0.5) were centrifuged (6000 *g*, 5 minutes), washed three times and resuspended in 200 µL PBS buffer (137 mM NaCl; 2.7 mM KCl; 10 mM Na_2_HPO_4_; 1.8 mM KH_2_PO_4_ in 1 L distilled water, pH adjusted to 7.4 with HCl; filtered). These cell suspensions were deposited in a glass 8-wells chamber (Ibidi, Martinsried, Germany) treated with poly-L-lysine (Sigma, Missouri, USA) for better adherence of living yeast cells. The incubation with 5 µM Laurdan (Sigma, Missouri, USA) was performed at room temperature for 1 hour followed by two washing steps with PBS. Images were obtained by two-photon excitation fluorescence microscopy on a Leica TCS SP5 (Leica Microsystems CMS GmbH, Manheim, Germany) inverted confocal microscope (DMI600), using a Ti:sapphire laser (Mai Tai, Spectra-Physics, Darmstadt, Germany) as the excitation source, and an apochromatic water immersion objective (63x, NA 1.2; Zeiss, Jena, Germany). The excitation wavelength was set to 780 nm and the fluorescence emission of Laurdan was collected at 400–460 nm and 470–530 nm. Laurdan exhibits shifts in its fluorescence emission maximum in response to changes in membrane hydration and solvent relaxation and this shift can be estimated through a normalized ratiometric measurement of the fluorescence intensity recorded in the two spectral channels, the generalized polarization (GP) value^56,81^. GP images were obtained and analysed using homemade software developed in a MATLAB environment (Mathworks, Natick, MA), with the GP value defined as GP = (I_400–460_ − G·I_470–530_)/(I_400–460_ + G·I_470–530_). Both channel intensities are corrected for background contributions, and the calibration factor G was obtained from imaging Laurdan in DMSO using the same experimental conditions as those set for the samples under study^82^.

### Permeability and plasma membrane potential estimation

Differences in the plasma membrane potential of the parental strain BY4741 and the derived deletion mutant *pdr18*Δ grown in the presence or absence of acetic acid were estimated based on the shift in the maximum emission wavelength of the fluorescent probe DiS-C_3_(3) (3-3′-Dipropylthiacarbocyanine iodide), as described before^83^. For the timepoints tested, yeast cells were harvested as described above for other assessments and resuspended in citrate-phosphate (CP) buffer to a final OD_600nm_ of 0.5. 5-mL aliquots of cell suspensions were labelled with DiS-C_3_(3) (Sigma-Aldrich) at a final concentration of 10^−7^ M, for 30 minutes at room temperature with orbital agitation of 70 rpm. Carbonyl cyanide *p*-chlorophenylhydrazone (CCCP) was added to an exponentially-growing culture of parental strain cells, to a final concentration of 5 µM, and this condition was used as a positive control for full membrane depolarization^84^. Fluorescence emission spectra of the cell suspensions were collected from 555 to 790 nm (at wavelength excitation of 531 nm) in 0.5 cm × 0.5 cm quartz cuvettes using a SLM-Aminco 8110 Series 2 spectrofluorometer (Rochester, New York, USA).

Plasma membrane permeability was assessed by the passive uptake of propidium iodide (PI; 20 mM in DMSO, Invitrogen), from cells harvested at adequate time points of the cultivation of parental strain and *pdr18*Δ cells either in the presence of absence of 60 mM acetic acid. PI was added to 1 mL of 4 × 10^7^ cells/mL to a final concentration of 20 µM, cell suspensions and incubated in the dark with orbital agitation (15 minutes, 250 rpm). Cells exposed to PI were centrifuged (17,500 × g for 5 minutes), washed twice and ressuspended in PBS buffer to a final 10^7^ cells/mL aliquots. PI-fluorescence emission spectrum (collected in the range 563–700 nm, at an excitation wavelength of 535 nm) were obtained using a SLM-Aminco 8100 series 2 spectrofluorometer with double excitation and emission monochromators MC400 (Spectronic Instruments, Inc., Rochester, NY), in a right-angle geometry. The light source was a 450 W Xe arc lamp and the reference a Rhodamine B quantum counter solution. All measurements were performed in 0.5 cm × 0.5 cm quartz cuvettes and under a constant temperature of 30 °C by using a thermostated sample holder with a Julabo circulating water bath (Houston, Texas, USA). Gain adjustment of the apparatus was performed using a positive control for full permeabilization (exponentially-growing parental strain cells exposed during 30 minutes to 50% ethanol in PBS). The spectra obtained were integrated and the area under the curve was calculated.

To evaluate the population heterogeneity for the cell permeability trait, at adequate time points the cell population strained with PI was observed using an Axioplan microscope equipped with adequate epifluorescence interface filters (BP450-490 and LP520; Zeiss). Fluorescence images were obtained with a cooled charge-coupled device camera (Cool SNAPFX; Roper Scientific Photometrics), and the images were analyzed with MetaMorph, version 3.5. Cell-to-cell fluorescence intensity was defined as the average of pixel by pixel intensity in the selected region of interest and a minimum of 50 cells/experiment were used. The fluorescence images were background corrected by using dark-current images.

### Data availability

All data generated or analysed during this study are included in this published article (and its Supplementary Information files).

## Electronic supplementary material

Supplementary Material
