# Supplementary material for: Pdr18 is involved in yeast response to acetic acid stress counteracting the decrease of plasma membrane ergosterol content and order
Source: Sci Rep. 2018 May 18;8:7860. doi: 10.1038/s41598-018-26128-7 (PMC5959924; doi:10.1038/s41598-018-26128-7)

Supplementary Material

Pdr18 is involved in yeast response to acetic acid stress counteracting the decrease of plasma membrane ergosterol content and order

Cláudia P. Godinho, Catarina S. Prata, Sandra N. Pinto, Carlos Cardoso, Narcisa M. Bandarra, Fábio Fernandes, Isabel Sá-Correia*

***Corresponding author:** isacorreia@tecnico.ulisboa.pt

Supplementary Table S1. Primers used for qRT-PCR analysis.

Target gene	Sequence (5'-3')
ACT1	fw: CTCCACCACTGCTGAAAGAGAA<br>rev: CCAAGGCGACGTAACATAGTTTT
PDR18	fw: TTGGCAAGCCGGATCTGT<br>rev: CCACGCGGATTGGGAAT
ERG2	fw: TTCCTTTGCCCTTGAATTGG<br>rev: AAACCCGAATGGCAACATACA
ERG3	fw: GCTCTGCACAAGCCTCATCA<br>rev: GGAAAGAATGAGATGCGAAAGG
ERG4	fw: CAACTCGGTGTTCCCATGGT<br>rev: AAGGCTCTGTGAATCAGGACAAC
ERG6	fw: GCTGGTATTCAAAGAGGCGATTT<br>rev: GCTGGGCCCCCAACA
ERG24	fw: GAATTGGGATGGGTGAAAGTT<br>ev: TGGAAGATGTGGAAACCCAAA

Supplementary Figure S1. Biological replicates of the growth curves of the parental (○, ●) and *pdr18*Δ (□, ■) strains in MM4 liquid medium supplemented (●, ■) or not (○, □) with 60 mM acetic acid at pH 4.0 based on culture OD_{600nm}.

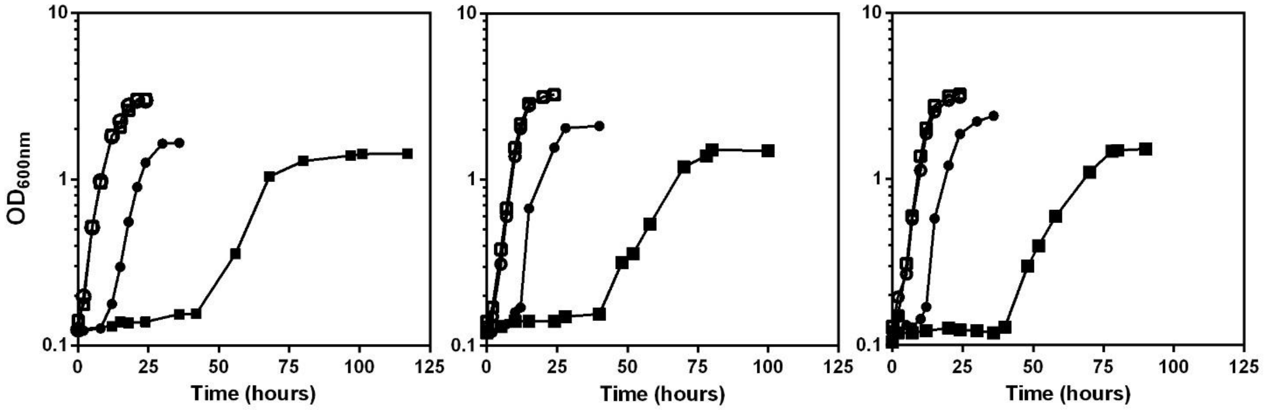

Supplement: Supplementary file 1 — Supplementary Material [file 41598_2018_26128_MOESM1_ESM.pdf]
